# Playing for a Healthy Life: Integrating Mobile Exergames in Physical Education

**DOI:** 10.3390/bs15020229

**Published:** 2025-02-18

**Authors:** Pablo Sotoca-Orgaz, Marta Arévalo-Baeza, José A. Navia

**Affiliations:** Faculty of Medicine and Health Sciences, University of Alcalá, 28805 Alcalá de Henares, Spain; marta.arevalo@uah.es (M.A.-B.); jose.navia@uah.es (J.A.N.)

**Keywords:** exergames, physical education, game-based learning

## Abstract

This study aimed to promote a coherent pedagogical framework for integrating mobile exergames into physical education (PE) as a strategy to reduce sedentary behavior. The intervention was grounded in the game-based learning methodology, assessing the impact of exergame practice on the physical and mental well-being of prospective PE teachers. The Borg Rating of Perceived Exertion (RPE) scale and a mental effort scale were used to evaluate perceived exertion across various mini-games, measuring physical intensity, motor engagement, and mental effort with participation from 130 undergraduate students in Physical Activity and Sport Sciences. Additionally, the pedagogical and motivational aspects of the Active Arcade v3.11 video game were analyzed to support its future integration into secondary education PE classes. Participants reported high levels of motor engagement throughout the program, accompanied by moderate physical intensity. They also emphasized the user-friendly nature of these augmented reality exergames and expressed enjoyment during the sessions. The findings suggest that mobile exergames hold considerable potential for enhancing skill acquisition and fundamental motor skills while promoting healthy habits among students in PE classes.

## 1. Introduction

The emergence of exergames has introduced a revolutionary concept in video gaming, requiring physical exercise as a mode of interaction (Medeiros et al., 2017; Staiano & Calvert, 2011). In these games, body movement serves as the primary form of engagement (Gómez-Miguel & Calderón-Gómez, 2023). Exergames are defined as video games “that promote (either via using or requiring) players’ physical movements (exertion) that is generally more than sedentary and includes strength, balance, and flexibility activities” (Oh & Yang, 2010, p. 9). These active video games represent a new generation of electronic entertainment that challenges the traditional view of passive gaming (Beltrán-Carrillo et al., 2011; Lozano-Sánchez et al., 2019), often criticized for promoting sedentary behaviors by replacing physical activity with prolonged use of computers, video consoles, and mobile devices (Castro-Sánchez et al., 2015), a phenomenon known as “technological sedentarism” (Castro-Sánchez et al., 2015).

This growing concern, highlighted by the World Health Organization (WHO), has prompted physical education professionals to develop innovative strategies to combat sedentary lifestyles. Excessive screen time, particularly among young people, is linked to rising obesity rates and other health issues stemming from insufficient physical activity (Laurson et al., 2008; Shen et al., 2021). In response, the WHO recommended the use of exergames during the COVID-19 quarantine in 2020 to reduce inactivity at home and promote healthy habits (World Health Organization, 2020). Since then, exergames has become increasingly popular as an alternative form of exercise (Luarn et al., 2023; O’Loughlin et al., 2023).

Exergames are designed to encourage movement-based interaction, encompassing a wide range of activities such as sports, active games, and dance (Cerezo-Pizarro et al., 2023). While some require consoles, screens, and platforms, technological advancements have facilitated the development of free-to-play exergames that utilize mobile devices with motion detection (Moller et al., 2023) and augmented reality (Marin-Suelves et al., 2022). This innovation allows users to engage in physical activity in virtually any space, including outdoors (Moller et al., 2023; Marin-Suelves et al., 2022), reflecting a growing preference for portable, multifunctional devices. Mobile phones, once primarily used for passive activities such as chatting or watching movies, now serve as convenient tools for active gaming.

In recent decades, mobile applications offering free-to-play games have emerged as a dominant form of global entertainment. Mobile phones, now widely accessible, are multifunctional tools for communication, expression, leisure, and information (Chóliz et al., 2009). One key motivation for adopting these technologies, particularly among adolescents, is their high entertainment value. Research shows that 94.8% of 15-year-olds in Spain own and use a mobile phone (Spanish Statistical Office, 2023), and 34% of adolescents engage in online gaming daily or almost daily during their leisure time (Hooft-Graafland, 2018). Given adolescents’ substantial engagement with mobile applications (Chóliz et al., 2009), mobile exergames present a promising strategy for increasing physical activity levels.

The popularity of active video games has surged over the past decade (Park et al., 2019), with research expanding into fields such as public health, rehabilitation, psychology, neuroscience, and computer science (Benzing & Schmidt, 2018; Gómez-Gonzalvo et al., 2018; Marin-Suelves et al., 2022). Within the educational sector, particularly in physical education, research on exergames has grown considerably (Gómez-Gonzalvo et al., 2018; Marin-Suelves et al., 2022). The pursuit of enhancing learning through active methodologies and the integration of educational technological resources in the classroom has driven novel strategies such as game-based learning (Pellas & Mystakidis, 2020). The proper use of game based learning has been proved to facilitate the assimilation of academic content, as well as the improvement of competencies and skills through a digital game (Pellas & Mystakidis, 2020; Qian & Clark, 2016). Studies suggest that exergames positively impact physical (Ho et al., 2022; Larsen et al., 2013), mental (Andrade et al., 2019; Lee et al., 2017), and social development (Li et al., 2018), while serving as a motivating tool for students (Lyons et al., 2014).

Various exergames have been evaluated for their impact on parameters such as caloric expenditure (Canabrava et al., 2018), motor skill improvement (Comeras-Chueca et al., 2022), cognitive function (Best, 2012), and activity enjoyment (Gu et al., 2023; Moholdt et al., 2017). These games often require full-body involvement, with findings indicating that muscle engagement and cardiovascular demands can meet recommended energy expenditure levels (Haddock et al., 2008; Siegel et al., 2009).

However, in classroom settings, measuring complicated parameters such as heart rate or accessing advanced platforms can be challenging for teachers. Therefore, pre-service physical education teachers would benefit from experience with free-to-play exergames, which require only a mobile phone with a camera and an application to track body movements. For example, the Active Arcade v.3.11 app, used in this study, is freely available. Nonetheless, the presence of digital resources alone does not guarantee skill development. Thus, integrating free-to-play exergames into physical education requires pre-service teachers to be trained (Marin-Suelves et al., 2022) to select, design, facilitate, and adapt these tools to the pedagogical contexts (Monguillot et al., 2018).

Before outlining the study’s objectives, it is essential to define key concepts such as motor engagement, practice time, perceived physical and mental effort, and motivational factors in motor activities. These variables are critical for evaluating the effectiveness of exergames in promoting health and well-being. Research consistently shows that sufficient motor engagement time and adequate intensity can significantly enhance physical and mental health (Martínez-Gómez et al., 2007). Motor engagement refers to the time students are actively involved in physical activities during PE classes or other movement-based activities, excluding time spent on organization, instruction, or waiting (Corpas-Ruiz & Villodres, 2023). Practice time denotes the period during which students perform specific motor tasks (Rodríguez-Reyes et al., 2020). Physiological engagement, defined as the time spent working at intensities high enough to induce organic adaptations, maximizes the health benefits of PE classes (Martínez-Gómez et al., 2007). Although various factors influence this relationship, effort can be measured subjectively through physical and mental exertion perception, allowing students to self-assess their engagement (Ouwehand et al., 2021; Rewitz et al., 2024). In addition, motivation and enjoyment are fundamental in achieving long-term benefits through physical activity (Fierro-Suero et al., 2023).

In summary, this study aims to assess the impact of a physical conditioning session using the mobile exergame Active Arcade v3.11 with future PE teachers. The study evaluated participants’ motor engagement and perceived effort, along with the app’s didactic, motivational, and user-friendly aspects. The following hypotheses were formulated: (i) Active Arcade v3.11 allows each individual to regulate their exerted effort which results in an adjusted perception of physical and mental effort according to their regular fitness; (ii) motor engagement will be significant and suitable for a 60 min session; and (iii) the levels of motivation and enjoyment generated will be high.

## 2. Materials and Methods

### 2.1. Participants

A total of 130 students, aged between 18 and 50 years (M = 19.31, MDn = 18, SD = 3.11) were selected. Of these, 68% (n = 89) were male and 32% (n = 41) female. All participants were enrolled in the cross-disciplinary course *Technology and Physical Activity*, a first-year requirement in the Bachelor’s Degree in Physical Activity and Sports Sciences at the University of Alcalá, Spain.

Inclusion criteria were as follows: attendance during the preliminary intervention explanations, absence of injuries on the day of the exergame session, and completion of informed consent and data collection questionnaires. The study was approved by the Research and Animal Experimentation Ethics Committee of the University of Alcalá (protocol code CEIP/2024/6/122).

### 2.2. Procedure

This study forms part of the Teaching Innovation Project titled “Proposals for Game-Based Learning through Exergames and Modern Board Games to Promote Motor Engagement” (Ref. UAH/EV1469) at the University of Alcalá. Before data collection, participants attended a 90 min in-person training session that introduced technologies such as augmented reality (AR) and outlined the research protocol and classroom procedures for the upcoming session.

**Figure 1:**
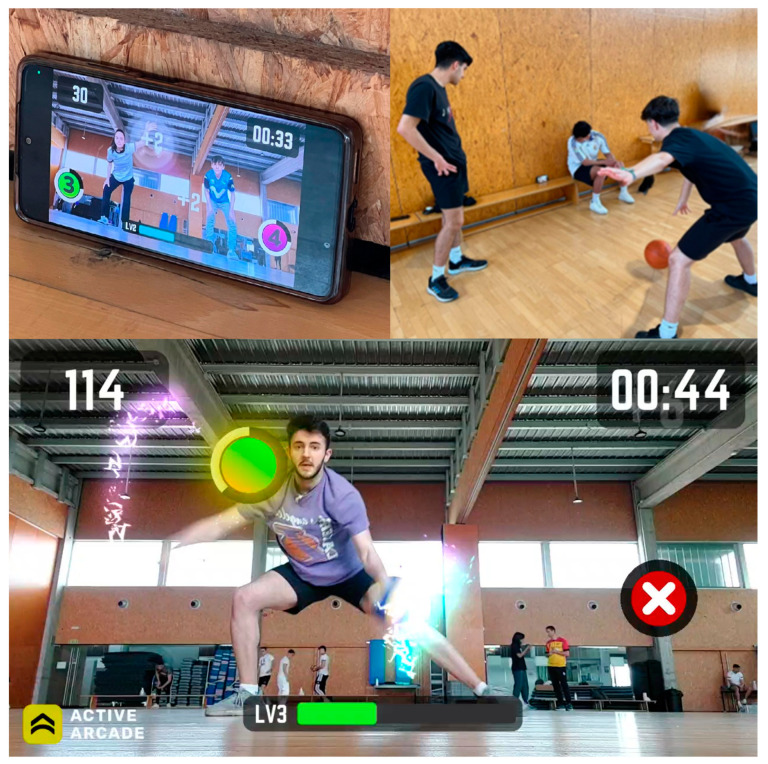
Students playing Active Arcade.

The practical session, conducted the following day, involved a 90 min Active Arcade v3.11 tournament at the university’s sports facilities. Students were divided into groups of 3 to 4, each equipped with personal devices (tablets or smartphones) with the Active Arcade v3.11 app installed. The instructor introduced each mini-game, allocating 12 min per game and a designated space for each group. After each mini-game, participants recorded their highest scores, identified the winning team, and reviewed performance results. The session concluded with certificates awarded to the highest scorers of each mini-game (Figure 1).

Following the tournament, participants completed an online questionnaire with close and open-ended questions addressing impact and their perceptions of exergames as future physical education teachers in secondary education. The questionnaire data were entered into an Excel database for further analysis.

#### Game Selection

The exergame app used in this study was Active Arcade v3.11 (NEX Team Inc., San Jose, CA, USA), released in 2021 and available for free on iOS and Android platforms without the need for registration. Active Arcade v3.11 employs motion recognition through the device’s camera, enabling users to interact with augmented holographic visuals and sounds via AR technology. This setup requires no peripheral sensors or wearable devices, only an internet connection via Wi-Fi or mobile data.

Active Arcade v3.11 offers 12 mini-games with individual and collaborative modes across multiple difficulty levels. Each mini-game starts with player body recognition through the front camera, initiating the gameplay screen. The primary objective is to perform physical movements to achieve the highest score within a set time limit. Players can review top scores and past gameplay in video format to assess performance accuracy.

Out of the 12 mini-games, six were selected for this study. Each mini-game was repeated up to four times, encouraging participants to improve their scores, compete for the top three class positions, and aim for the tournament record. The selection was made collaboratively by course instructors, who are experts in Physical Activity and Sports Sciences with extensive experience in playful methodologies. The selection was based on four criteria:

- Transferability to school physical education: fostering motor skills development (e.g., movement, jumping, throwing, catching) and enhancing physical attributes such as speed, endurance, and strength.
- Session timing: appropriate intensity level categorized into warm-up, main activity, or cool-down phases.
- Difficulty level: assessed by the application (beginner, intermediate, or advanced) and required space (large or small).
- Game type: gameplay mechanics, competitive or cooperative modes, and individual or team-based play.

Based on these criteria, the following mini-games were selected:

- Cone Knockout (Intermediate Level: Warm-Up Phase)
  Individual agility and speed endurance game. Duration: 60 s.
- Reaction Flow (Beginner Level: Warm-Up Phase)
  Reaction speed game played in pairs. Duration: 60 s.
- DribbleTag (Advanced Level: Main Activity Phase)
  Individual reaction speed and coordination game involving basketball dribbling and passing. Duration: 60 s.
- Box Attack (Advanced Level: Main Activity Phase)
  Individual reaction speed and strength game. Duration: 60 s.
- Galaxy Jumpers (Beginner Level: Cool-Down Phase)
  Team-based jumping game (3–4 players). Duration: until the last player remains active.
- SuperHits (Intermediate Level: Cool-Down Phase)
  Individual rhythm game. Duration: the length of the chosen song.

Certain mini-games were excluded to avoid redundancy:

▪ High-Kicks and Reaction were omitted due to similarities with Reaction Flow.
▪ Bunny Hop, Whack a Mole, Fit Pals, and Space Pong were excluded due to their resemblance to Galaxy Jumpers or SuperHits.
▪ Pose was excluded as it focuses on expressive movement rather than physical exertion.

### 2.3. Instruments

A questionnaire was developed to assess perceived exertion during the mini-game sessions, utilizing the Borg CR10 Scale, a validated tool for subjective evaluation of physical exertion on a 0–10 scale, where 0 represents minimal exertion and 10 denotes maximum effort (Williams, 2017). This scale was specifically used to measure motor engagement throughout the session. To evaluate mental effort perception, a pictorial scale from similar tools was employed (Ouwehand et al., 2021), corresponding to a 0–10 Likert scale to ensure consistency with the Borg CR-10 Scale results for subsequent analysis.

Building on prior research on digital leisure and exergames in physical education (Chacón-Cuberos et al., 2016), open-ended questions were added to explore participants’ familiarity with active video games by asking how frequently they played on digital devices, their experience with video games in the classroom, and their opinion about the potential for integrating these tools into PE curricula. Additionally, consistent with related studies (Hoyos-Quintero et al., 2022), data were collected on participants’ frequency of physical exercise during the week, the type of activity performed, and its intensity, using the Borg CR-10 Scale as a reference.

### 2.4. Data Analysis

This study followed a mixed-methods approach. For the quantitative analysis, descriptive statistics were reported, including the mean (M), standard deviation (SD), and 95% confidence intervals (CIs 95%). Since repeated observations were collected from the same participants, a Repeated Measures Analysis of Variance (RM ANOVA) was conducted. Tukey’s post hoc test with adjusted *p*-values was used for pairwise comparisons. When applicable, an interparticipant factor was added to the model to examine the influence of gender and practice frequency on the measures. Error bars in the ANOVA figures represent the confidence intervals. Effect size was measured using partial eta squared (η_p_^2^), with values of 0.01, 0.06, and 0.14 indicating small, medium, and large effects, respectively. Additionally, Pearson’s *r* test was used to assess correlations between intensity measures.

For the qualitative analysis, Atlas.ti (v.9.0) was used to examine open-ended responses through thematic and categorical analysis, identifying patterns of meaning in the data (Braun & Clarke, 2006). Based on the coding of responses, the following categories and subcategories were generated, allowing for the identification of broader themes for interpretation: (1) Ease of Use and Potential Applications in the PE Classroom: Opportunities and Limitations, (2) Exergames as Promoters of Motor Skills, (3) Motor Engagement as Effective Practice Time, and (4) Motivation and Enjoyment.

## 3. Results

To facilitate the analysis of the questionnaire results, responses were organized according to question type. First, data from quantitative questions were presented to provide a structured overview of numerical findings. Subsequently, these results were complemented by an analysis of open-ended questions, examined through a qualitative lens to capture participants’ perspectives in greater depth.

### 3.1. Quantitative Measures

After the session, the measured levels were:

- Physical intensity: *M* = 6.55 [CI 95% 6.27–6.82] *SD* = 1.58.
- Mental intensity: *M* = 5.63 [5.28–5.98] *SD* = 2.04.
- Motor engagement *M* = 7.32 [7.03–7.60] *SD* = 1.67.

**Figure 2:**
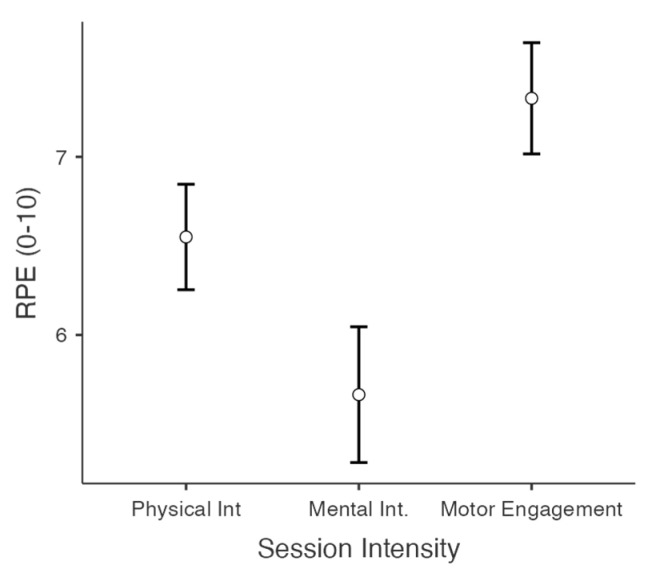
Reported intensity scores of the aspects of the exergames’ practice.

ANOVA results revealed significant differences among these measures, F(2,258) = 64.87, *p* < 0.001, η_p_^2^ = 0.33 (Figure 2). Post hoc pairwise contrasts indicated that motor engagement during the session was significantly higher than physical and mental intensity (*p* < 0.01), and physical intensity exceeded mental intensity (*p* < 0.001).

**Figure 3:**
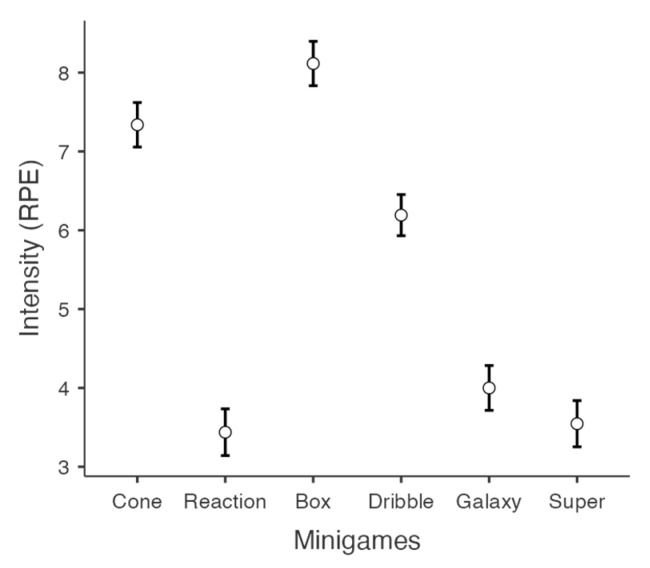
Reported intensity scores of the selected Active Arcade mini-games.

As depicted in Figure 3, mini-games also differed in intensity, F(5,645) = 279.40, *p* < 0.001, η_p_^2^ = 0.68. Specifically, Box Attack and Cone Knockout elicited intensities exceeding 7 points, whereas Reaction Flow, Galaxy Jumpers, and SuperHits showed relatively lower intensities (Figure 3). Post hoc pairwise differences among mini-games are detailed in Table 1.

The reported intensity of the mini-games was positively correlated with physical and mental intensities, as well as motor engagement during the session (Table 2). For instance, participants who perceived high intensity during the Box Attack game also reported high levels of physical, mental, and motor engagement. Interestingly, motor engagement was found to be more closely related to mini-game intensity than either physical or mental exertion.

**Figure 4:**
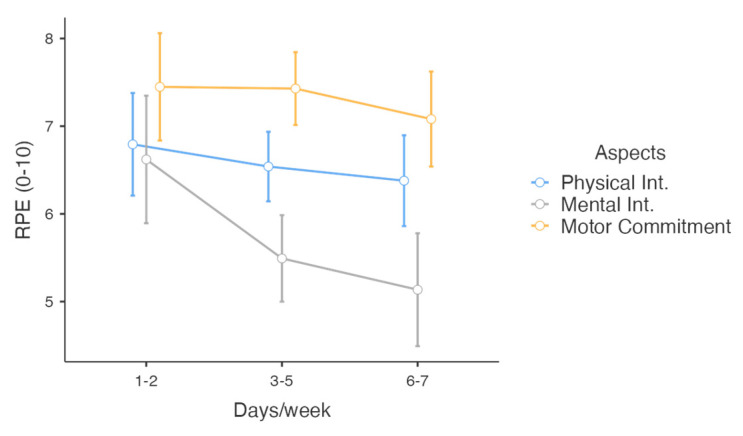
Session’s intensities by frequency of practice.

All participants reported engaging in regular weekly sport practice: 22% (n = 29) practiced 1–2 days per week, 49% (n = 63) practiced 3–5 days per week, and 29% (n = 37) practiced 6–7 days per week. The average perceived intensity of their training days was *M* = 7.16 [6.97–7.35] *SD* = 1.08. A repeated measures ANOVA (RM ANOVA) revealed a significant interaction between the number of practice days and the session’s overall intensity, F (4,252) = 3.02, *p* = 0.018, η_p_^2^ = 0.05. This interaction was primarily driven by mental intensity (Figure 4). Unlike physical intensity or motor engagement, mental intensity was significantly influenced by the frequency of physical practice, F(2,126) = 4.95, *p* = 0.009, η_p_^2^ = 0.07 (Figure 4). Participants practicing more than two days per week reported lower mental effort compared to those practicing 1–2 days per week (post hoc *p* < 0.05). Finally, no significant main effects or interactions were found related to gender (all *p* > 0.05), session practice intensities (physical, mental, and motor engagement), mini-game intensities, regular practice frequency, or overall session intensity.

### 3.2. Qualitative Measures

The following section presents the themes identified from the questionnaire responses. The testimonies included are among the most representative, illustrating the most recurrent patterns observed in the analysis of the responses.

#### 3.2.1. Ease of Use and Potential Applications in the PE Classroom: Opportunities and Limitations

Many participants emphasized the importance of integrating exergames like Active Arcade v3.11 into PE classes, noting the potential for coherent technology integration in PE. For instance, E71 stated, “mobile phones should always serve pedagogical purposes in the classroom”. Similarly, E14 highlighted that “this type of content is not typically presented in this way in high schools”, while E58 pointed out how it can foster “a healthy relationship with technology”.

Regarding technical assessment, the application was praised for its intuitive functionality and usability. E107 described it as “intuitive and easy to use”, and E22 appreciated the convenience of not needing additional equipment, noting that “it fits in one’s pocket”. E121 further emphasized its accessibility: “everyone has a mobile phone they usually bring to class, so everyone can download the game and use it”. Additionally, E44 noted that the application is widely available as it “can be downloaded on any device, as it is a free resource accessible on both Android and iOS systems”.

#### 3.2.2. Exergames as Promoters of Motor Skills

Participants associated the use of Active Arcade with developing motor skills and enhancing physical fitness. E90 noted, “in nearly all the games, you’re moving in different ways and have to do so quickly; in others, you jump, and in the basketball game, you control a ball through dribbling while maintaining a continuous bounce”. E75 observed, “in the box game, lower-body strength exercises, such as jumps, are combined with upper-body exercises like push-ups”. Several participants emphasized the games’ speed and reaction requirements. E23 explained, “they are all speed games because you have a limited time to achieve the highest score, and some require reaction time as you don’t know where on the screen the elements you need to tap will appear”.

Moreover, the games were found to increase enjoyment and participation. E55 remarked, “when exercises like jumps, push-ups, or speed exercises are incorporated into games, they become much more enjoyable and encourage participation without reluctance”. This sentiment was echoed by E44, who highlighted the games’ versatility beyond PE: “this app can also be used in training sessions. Personally, I’m now considering incorporating it into my basketball practice design at my club”.

#### 3.2.3. Motor Engagement as Effective Practice Time

Participants noted high activity levels throughout the session. As E23 explained, “we only rested when it was our teammates’ turn”. Some admitted finding ways to extend their playtime, such as E33, who said, “we sometimes used two smartphones to play longer”. The 60 s duration of most challenges encouraged continuous activity with minimal downtime. E15 remarked, “since the rounds didn’t last long and mini-games changed every 15 min or so, we tried to maximize our time by using two or three phones to play and achieve high scores”. The structure enabled participants to repeat challenges multiple times. E01 noted, “we completed each challenge about 10 times”. E11 highlighted the intensity: “these short, intense series make you want to give your maximum effort, and when you’re really focused, class time flies by”. Some participants acknowledged taking breaks during game rotations or instructions, while others admitted to stopping due to fatigue. E15 observed that “it seemed to be a low-intensity session, but we were moving for over an hour”, while E45 observed, “each person set their own intensity level”, with some noting that “there were also more relaxed games at the end of the session” (E18).

#### 3.2.4. Motivation and Enjoyment

Participants frequently highlighted the motivational aspect of integrating video games into physical activity. E74 described it as “intriguing and appealing, making it possible to exercise through video games in the classroom”. E65 remarked that this approach “redefines the notion of video games, shifting from sedentary play to active engagement”. Participants acknowledged its potential for promoting “healthy leisure” (E92). E103 observed, “you play at an intense level, as reflected by your heart rate; it’s exercising without realizing it”. E23 added, “if taken seriously, the intensity of many games rivals traditional cardio or strength training sessions”.

Competitive and cooperative dynamics also played a role. E67 highlighted the impact of extrinsic motivation on exercise intensity, observing that “Competition drives intensity, as people push to achieve high scores”. Meanwhile, E32 emphasized intrinsic motivation: “I didn’t focus on others’ top scores; I focused on improving my record, competing against myself”. Cooperative gameplay was also valued, with E22 noting, “Playing with others adds responsibility—you want to do your best”.

## 4. Discussion

This study evaluated the impact of a mobile exergame (Active Arcade v3.11) on motor skill engagement, perceived physical and mental exertion, and the motivational and usability aspects for training future PE teachers. Results align with previous research (Chow & Mann, 2023; Conde-Cortabitarte et al., 2020; Marin-Suelves et al., 2022; Staiano & Calvert, 2011), demonstrating the benefits of exergames in promoting healthy habits and improving physical, emotional, and cognitive outcomes.

Participants’ moderate-to-high weekly physical activity levels reflected their prior training experience, with no significant gender differences in perceived intensity and frequency. These values reflect the participants’ experience in physical activity training sessions. Participants reported similar levels of motor engagement accompanied by moderate levels of physical and mental exertion. Lower activity participants reported higher mental exertion, suggesting that cognitive and physical effort are distinct phenomena that require different adaptations (Cárdenas et al., 2015). Ultimately, Active Arcade v3.11 is a video game that can be used by students of any fitness level. The variety of mini-games targeted various skills and abilities—strength development (*Box Attack*), basic motor skills such as jumping and dodging (*Galaxy Jumpers*), reaction speed (*Reaction Flow*), coordination (*SuperHits*), and sport-specific skills (*DribbleTag*)—which highlights the value of this tool in a physical education classroom. This variety sustained high motor engagement without excessive physical demands, favouring practice density over intensity to maximize energy expenditure (Lyons et al., 2011; Sell et al., 2008).

This type of design may result in being more convenient for PE. Instead of higher levels of physical or mental load during PE classes, enough engagement time is necessary. It is not about the amount of intensity but the density of practice (Vaghetti et al., 2018). Additionally, the variety of mini-games promoted sustained engagement, offering a dynamic learning experience (Sheehan et al., 2015). The adequate selection of activities and the use of freely and easily available resources like free-to-play exergames maximize the benefits of physical activity sessions in terms of motor and physiological engagement (Martínez-Gómez et al., 2007). The accessible (Díaz-Barahona, 2020), cost-free nature of exergames (Dixon et al., 2010), combined with their user-friendly and playful approach, encouraged active participation, supporting their integration into PE curricula (Díaz-Barahona, 2020). There is ongoing debate and some controversy regarding the use of technology in educational settings (Wilmer et al., 2017). We, however, consider that its positive or negative impact mostly depends on how well technology is implemented and integrated into pedagogical processes (Fawns, 2022; Selwyn, 2016). In our specific case, mobile devices contributed to the improvement in the practice of physical exercise in a self-regulated and highly motivating manner.

From a motivational perspective, participants reported high enjoyment levels. According to the literature, game-based learning can enhance interest in physical activity (Huang et al., 2019; Marques et al., 2023). Despite concerns about the impact of video games on young people’s health and socialization (Gómez-Gonzalvo et al., 2020), an exergame like Active Arcade v3.11 reshapes perceptions of video games. Teachers (or future teachers) can effectively use exergames, with innovations like augmented reality (Omarov et al., 2024), as allies to combat sedentary behaviors among students (Dávila-Morán et al., 2024; Liu et al., 2020; Santos et al., 2021). As has been observed in other studies, motivational aspects, particularly competitive and cooperative elements, enhanced engagement, fostering a positive learning environment (Marker & Staiano, 2015).

Prior experience with digital tools is essential for integrating exergames into PE programs (Conde-Cortabitarte & Rodríguez-Hoyos, 2018). Research indicates that adults often underestimate the potential of technology to improve health outcomes until they encounter its benefits through exergames (Bird et al., 2015). This underscores the importance of training future PE teachers in the pedagogical use of these technologies (Díaz-Barahona, 2020; Meckbach et al., 2013; Naranjo-Harnisth, 2024). Such training fosters critical thinking skills and prepares educators to effectively integrate digital health applications (mHealth) (Fitzpatrick & Russell, 2013; Ubago et al., 2022).

The findings of this study further emphasize the possibility of incorporating technological applications and exergames into PE classrooms (Díaz-Barahona, 2020; Quintero-González et al., 2022; Vaghetti et al., 2018).

The current results indicate the perceived intensity of different exergames. In this regard, a direct practical implication would be that high-intensity mini-games (i.e., Box Attack and Cone Knockout) need to be placed in the middle of the session, whereas lower intensity ones (e.g., Reaction Flow, Galaxy Jumpers, and SuperHits) can be used as warm-up or cool-down exercises. Moreover, future teachers should explore the pedagogical potential of exergames to transform recreational video game use into meaningful educational experiences (Conde-Cortabitarte et al., 2024; Zhao et al., 2024). This intervention serves as a pilot training initiative for prospective PE teachers, enabling them to critically and professionally assess the educational impact of video games in classroom settings.

The results suggest that exergames, when implemented with thoughtful curricular planning, can serve as effective tools for PE instruction. These tools not only support motor skill development and the promotion of healthy habits (Dávila-Morán et al., 2024; Vernadakis et al., 2015), but also enhance enjoyment and motivation towards physical activity through innovative digital resources (Lyons et al., 2014; Marin-Suelves et al., 2022).

## 5. Conclusions

This study highlights several limitations and potential areas for further research. One major limitation is the current inability to collect real-time physiological fitness data without compromising usability. However, as smartphone technology evolves and heart rate sensors become more integrated, exergames are expected to offer more comprehensive data in the near future.

From a pedagogical standpoint, future research should explore how educators can effectively design and implement exergaming sessions, integrating them into structured lesson plans or extracurricular activities. The intensity levels reported in this study across different mini-games could serve for such planning. Additionally, further research should focus on equipping educators with the skills to incorporate these digital tools into educational environments. Exergames in PE classes can encourage young people to maintain sustained physical throughout their lives, serving as a motivating option that promotes movement in favor of their health.

In conclusion, free-to-play exergames, leveraging mobile devices and interactive games, hold substantial potential to inspire educators to adopt these tools in their teaching practices. Their user-friendliness, engaging nature, and adaptability make them an enjoyable and effective way for students to engage in physical activity, both inside and outside the context of physical education.

## Figures and Tables

**Table 1:** Tukey post hoc *p*-values of mini-game intensities.

	Reaction	Box Attack	Dribble	Galaxy	Super
Cone	<0.001	<0.001	<0.001	<0.001	<0.001
Reaction		<0.001	<0.001	0.007	0.986
Box Attack			<0.001	<0.001	<0.001
Dribble				<0.001	<0.001
Galaxy					0.088

**Table 2:** Pearson correlation matrix between mini-games’ intensities and session’s intensities.

Active Arcade Mini-Games	Physical Intensity		Mental Intensity		Motor Engagement	
Cone Knockout (RPE)	0.38	***	0.27	**	0.41	***
Reaction Flow (RPE)	0.17		0.10		0.17	
Box Attack (RPE)	0.41	***	0.33	***	0.43	***
Dribble Tag (RPE)	0.47	***	0.31	***	0.49	***
Galaxy Jumpers (RPE)	0.20	*	0.19	*	0.18	*
SuperHits: Catch and Slash (RPE)	0.25	**	0.15		0.23	**

Note. * *p* < 0.05, ** *p* < 0.01, *** *p* < 0.001.

## Data Availability

The original contributions presented in the study are included in the article; further inquiries can be directed to the corresponding author.
